# Investigation of Microstructure and Interfacial Reactions of Diffusion Bonding of Ni-Ti6Al4V Materials Joined by Using Ag Interlayer

**DOI:** 10.3390/ma17184462

**Published:** 2024-09-11

**Authors:** Şükrü Çetinkaya, Haluk Kejanli

**Affiliations:** Mechanical Department, Engineering Faculty, Dicle University, Diyarbakır 21280, Turkey; kejanlih@dicle.edu.tr

**Keywords:** Ni, Ni6Al4V, diffusion welding, metallurgical structure, SEM, EDS, X-ray

## Abstract

Due to its super plasticity, low weight, and high mechanical resistance properties, generally, Ti6Al4V is used for aeronautical applications. However, it has low resistance to plastic shearing. In addition, it has poor wear resistance. For these reasons, a lot of techniques have been developed to improve its wear resistance. Investigations of microstructure and interfacial reactions of diffusion bonding of Ni and Ti6Al4V materials have been performed experimentally. Ni samples were prepared with 50 ± 5 µm Ni powders in cylindrical shape. For diffusion bonding, Ag foil was used for improving the interlayer and connection quality. Nickel and its alloys can be joined by using some different processes, and the use of an interlayer can further facilitate the joining process and improve the joint quality. The experiments were carried out under the protected atmosphere. Argon gas was used for protection. The experiments were performed under 5 MPa pressure for 60 min duration at 850 °C, 900 °C, and 950 °C thermal conditions. Investigations of metallurgical structure occurring in the interface areas were examined by optic analysis of EDS, SEM, and X-ray. The strength of the joints was tested by lap-shear tests. From observations, the best quality of the coalescence at interfaces was indicated at elevated temperatures.

## 1. Introduction

The aim of this study is to examine the internal structure phase changes in Ni and Ti6A14V materials joined by the diffusion welding method using an Ag interlayer and to determine the effect of these phases on the mechanical properties of the materials.

Nickel is the critical metal in the stainless steel industry and it is widely used in traditional applications [1]. The aerospace industry’s continuous technological advancements need the development of materials that are appropriate for high-temperature operation. Superalloys based on nickel have excellent corrosion resistance and high thermal conductivity [2]. Ni-based alloys have been employed a lot in the past few years as matrix materials because of their good mechanical qualities and high-temperature thermal stability [3].

The comprehensive performance of Ti6Al4V makes it a popular choice in aerospace, defense, and biomedical industries [4,5]. Titanium alloys are highly regarded for their impressive performance as bone implants in medical procedures [6]. In particular, Ti6Al4V alloy is extensively utilized in the biomedical field due to its advantageous properties, including low density, high strength, low elastic modulus, good corrosion resistance, and excellent biocompatibility [7,8]. The development of technology and industry has increased the need for combining different types of materials, and this has been the subject of many studies [9]. However, to expand the application and usage areas, these components need to be combined to produce more complex shapes. Diffusion bonding is the joining of two parts in a solid state under high pressure and temperature in the close contact surface area. Since the grains from these two original parts combined form common edges along the original bond line, an undetectable original bond line is obtained. Diffusion bonding may be the most suitable welding method to join these components due to the low bonding process temperatures [10,11]. Melting and resolidification are two steps in the solid-state bonding process, known as diffusion bonding [12]. The development of diffusion bonding, titanium, and its alloys to other high-tech structural materials, such as magnesium, stainless steel, and aluminum, has been thoroughly examined by Cooke and Atieh [13].

The Ti6Al4V alloy is widely used in many industries due to its high engineering properties [14]. However, the high production costs of Ti-based parts, processing difficulties, low plastic slip resistance, and poor wear resistance significantly limit their usage areas [15,16]. Numerous techniques have been developed for increasing the wear resistance of Ti6Al4V alloy [17,18,19]. Ag alloy has some advantages of excellent thermal and good electrical conductivity, low and stable contact resistance, high arc ablation resistance, and anti-arc erosion in power switch and power chip connections, and it is thought that it can meet the new requirements of future industries for electric vehicles or aerospace technology materials [20,21,22,23]. It was discovered that as the content of Ni rose in samples soldered together, the adhesive strength of the connection also increased [24]. The solubility of Ag and Ni metals is poor in the solid state, and they are unable to form a brittle intermetallic at temperatures greater than Ni’s melting point (1453 °C) or merge into a single liquid phase [25,26]. The use of a suitable foil can help the formation of strengthening phases in the joint area [27,28].

It has been shown that better mechanical and electrical properties can be obtained by adding Ag as the intermediate layer of the connection in the diffusion joining of Ti6Al4V and nickel [29,30,31,32,33]. Furthermore, the diffusion mechanism’s foundation is distinct from that of diffusion layers, which are made up of mixtures of the Ti–Cu system’s intermetallic and silver phases. As a result, the thickness and makeup of these diffusion layers determine the mechanical properties, and these parameters vary with bonding time [34]. The mechanical behavior of the connections can be explained by identifying the intermetallic phases that occur in the bonding region; these phases are correlated with the bonding conditions that are used [35,36].

## 2. Materials and Methods

### 2.1. Preparation of Ni Samples

**Figure 1:**
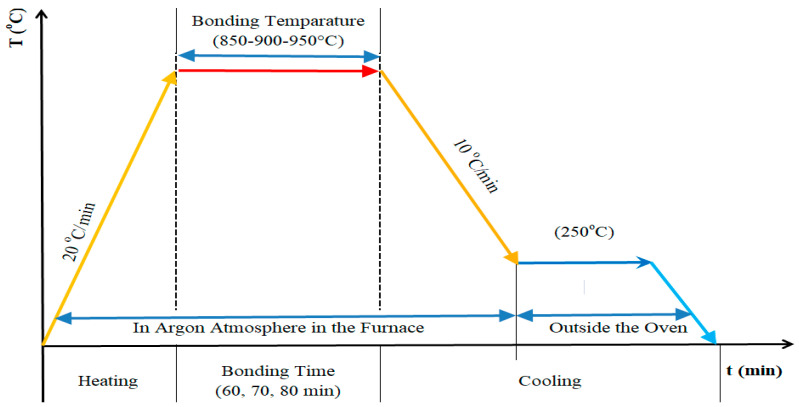
The sintering process of Ni samples.

Nickel samples were manufactured with pure nickel element powders with average dimensions of 50 µm in size. First, the powders were properly mixed with a mechanical mixer. Then, the mixture was cold compacted at 20 MPa pressure in the steel dies with the dimensions of 10 mm diameter and 11 mm height. And then it was sintered at 950 °C under an argon-protected atmosphere for a 60 min duration. The materials were kept under a 5 MPa uniaxial load for 60 min by applying 20 °C/min temperature increments. When the oven temperature decreased to 250 °C, the samples were taken out of the oven and left to cool at room temperature (Figure 1).

### 2.2. Diffusion Bonding of Ni-Ti6Al4V by Using the Ag Interlayer

**Figure 2:**
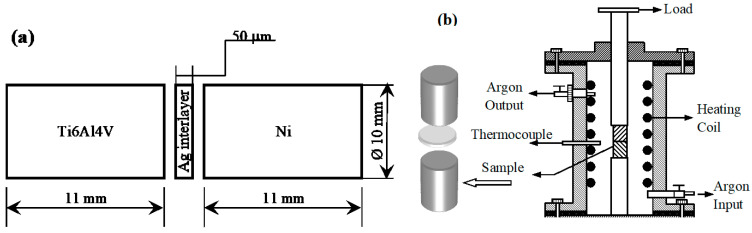
Schematic views of samples (**a**) and diffusion bonding machine (**b**).

Chemical contents of Ti6Al4V material is presented in Table 1. Test samples used in diffusion welding need to have their connection surfaces shielded from oxidation and corrosion. The test specimens were fused together using an Ag interlayer and the diffusion welding technique. As an interlayer (bonding aid), pure Ag (99.00%) with foil thickness of 50 µm and foil hardness of 120 HV was utilized to promote comparatively smooth and uniform surfaces and inhibit development of the brittle phases between powder metallurgy components. Parts were able to diffusely bind under continuous pressure for varying times and temperatures. The process of diffusion welding was carried out in an argon-protected environment for 60 min at 850, 900, and 950 °C under a constant pressure of 5 MPa (Table 2). A schematic depiction of the diffusion bonding equipment is presented in Figure 2.

### 2.3. Microstructure Analysis, Microhardness, and Lap-Shear Tests

**Figure 3:**
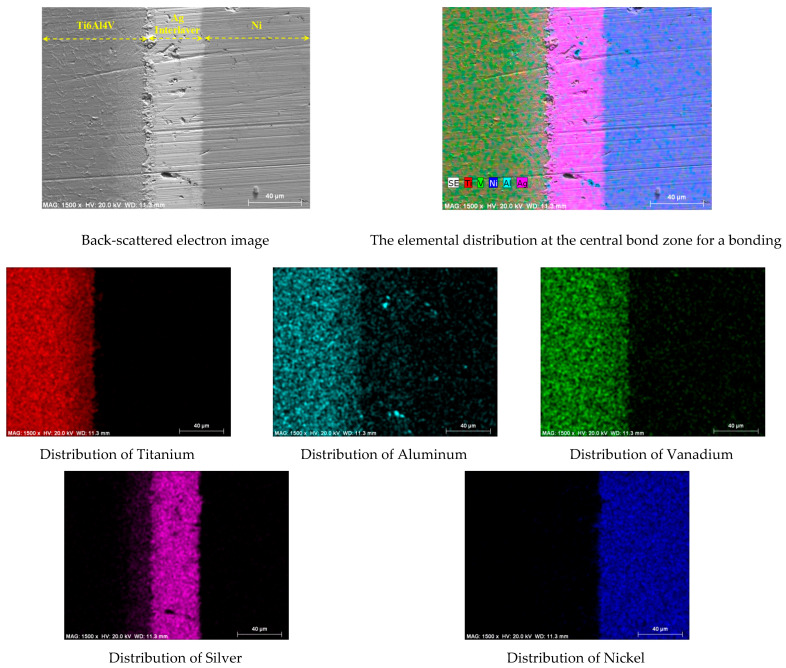
Backscattered electron images and elemental distribution.

The test specimens were sliced perpendicular to the bonding interface to make the longitudinal microstructure analyses easier. After the cutting surfaces were ground, they were etched for metallographic analyses using an etching reagent (2 mL HF, 10 mL HNO_3_, and 88 mL H_2_O). Optical microscopy, SEM, EDS, and X-ray analysis were used to help with the metallographic tests and assessments. Using a Leica MHF-10 test device and an HV scale on both sides, microhardness values in the diffusion couples’ interface area were determined over the course of 20 s while a 10 g load was applied (Figure 3).

To further investigate the interface microstructure, the sample assembled at 850 °C was carefully observed by SEM in backscattered electron mode. The SEM image of the samples, the distribution image of the silver interlayer in the main materials, and the mapping of different important elements are exhibited in Figure 3. It can be seen that the silver interlayer shows rich dispersion on the Ti6Al4V side.

**Figure 4:**
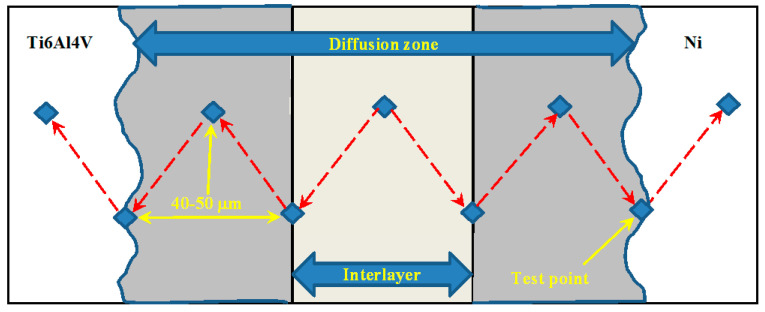
Location of microhardness measuring points and longitudinal spacing of the diagonal point of the notch.

**Figure 5:**
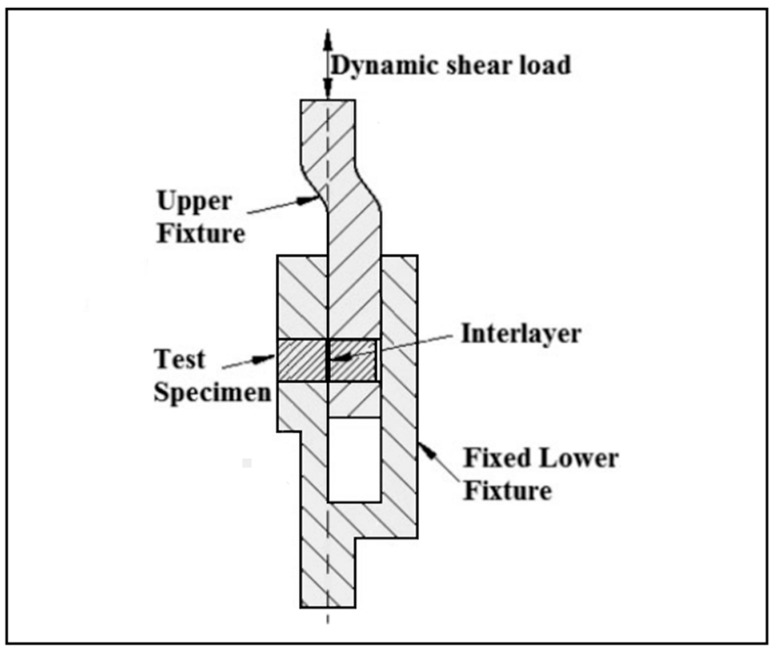
Schematic view of shear strength testing apparatus [9].

The location of microhardness measuring points are shown in Figure 4. Following this procedure, the specimens underwent a lap-shear test in accordance with ASTM D3165–07 standard. The schematic view of the lap-shear test apparatus is depicted in Figure 5.

In addition, in the contacted regions on both sides, possible phases of high-atomic-number nickel with silver were detected in the EDS analysis. Elemental maps show that the elemental distribution of low-atomic-number aluminum is more uniform than that of nickel.

## 3. Results and Discussion

### 3.1. Microstructure

Events in the metallurgical stages of the diffusion bonding can be enumerated as follows: deformation of surface roughness, plastic flow and creep, grain boundary diffusion of atoms to the voids, grain boundary migration, and volume diffusion of atoms to voids. Adherent surface oxides can facilitate diffusion bonding, particularly in Ni–Ti–Cu alloys. The oxide is generally not eliminated but, rather, spread over a larger surface area in a confined space where oxidation cannot occur again.

**Figure 6:**
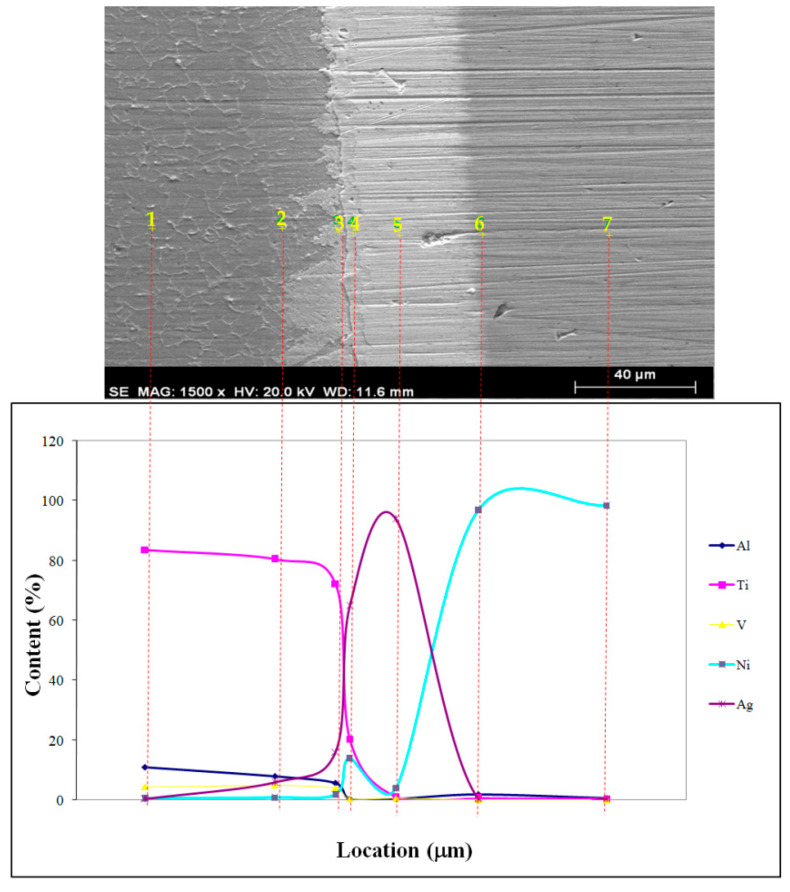
SEM microstructure and EDS analysis of different positions at 850 °C.

**Figure 7:**
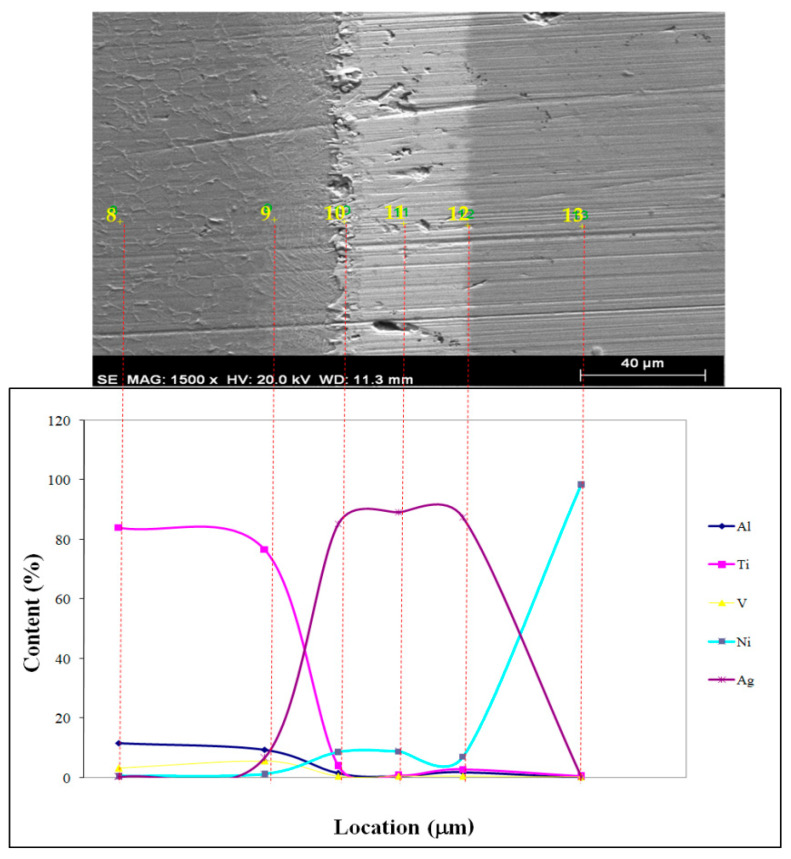
SEM microstructure and EDS analysis of different positions at 900 °C.

**Figure 8:**
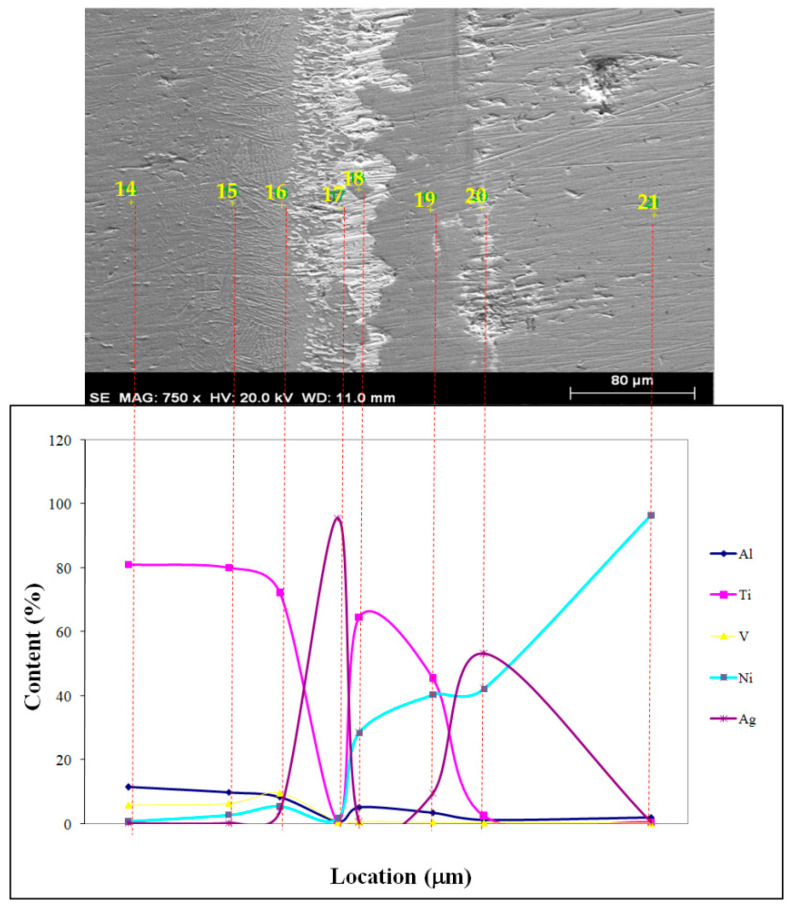
SEM microstructure and EDS analysis of different positions at 950 °C.

Insufficient interface diffusion, resulting from the low temperature and time, is depicted in Figure 6. EDS point scan results at different locations are given in Figure 6. To attain the same coalescence as depicted in Figure 6, diffusion mechanisms were increased and diffusion period was shortened at high temperatures. When the macro level specimens from the sharing experiment were analyzed, the fracture occurred sharply and there was a reasonably smooth surface free of plastic deformation. It is possible that sharp phase forms during temperature acceleration led to this condition. EDS point scan results at different locations are given in Figure 7 and Figure 8. Nearly all specimen fractures in SEM investigations happened at the joint borders between the primary material and the interlayer, as seen in Figure 7 and Figure 8. The numerical EDS spot scan results and corresponding possible phases of the samples at different temperatures (850, 900, and 950 °C) are given in Table 3, Table 4 and Table 5.

### 3.2. Microhardness

**Figure 9:**
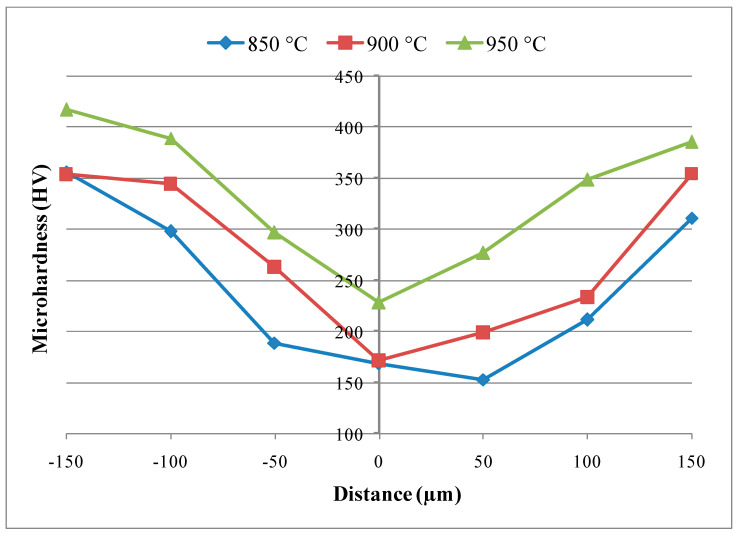
Microhardness graphic of the specimens N1 (850 °C), N2 (900 °C), and N3 (950 °C).

Figure 9 shows the distributions of microhardness at bonding contacts. There was an average of seven hardness metrics. The hardness value of the interface generally declined in all specimens, as shown by the figures. The values of microhardness increased at both sides of the bond contact. There was migration of alloying elements, including aluminum, nickel, and titanium, towards the interface region, which resulted in an increase in intermetallic phases and immediate hardness. The diffusion impact of the procedure resulted in low hardness zones on the Ni-Ti6Al4V side 20 mm from the contact.

### 3.3. EDS and X-ray Diffraction Analysis

**Figure 10:**
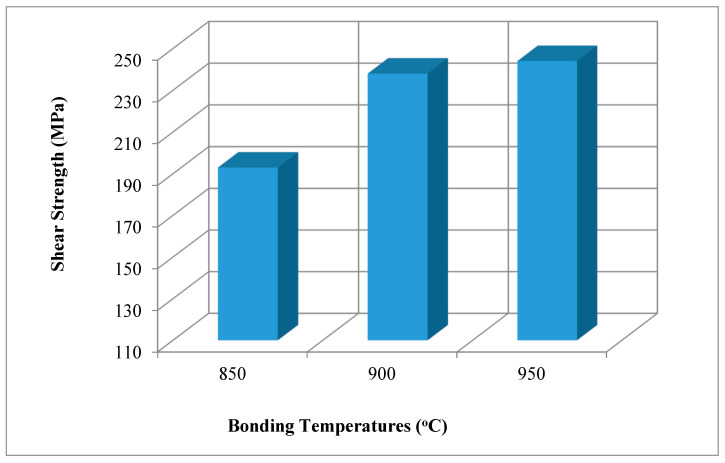
Lap-shear strength temperature graph for the specimens.

EDS, SEM, and X-ray analyses were performed to examine the mutual diffusion of nickel, titanium, aluminum, and vanadium atoms at both sides of interfaces. The results are shown in Figure 10 for a process temperature of 950 °C. The quantities of Ni at 8–13 µm from the Ti6Al4V side surface were measured at 850, 900, and 950 °C. Once more, at 850, 900, and 950 °C, Ti, Al, V, and Ag elements departed from the interface at the nickel composite at a distance of 8–13 µm. It showed that when welding temperatures rose, so too did the mutual migration and diffusion of Ni, Ti, Al, V, and Ag atoms.

### 3.4. The Lap-Shear Test

The resistance of Ti6Al4V and Ni pairs formed at various temperatures, employing a silver interlayer against the force of the diffusion source, was measured using the lap-shear tests. Figure 10 displays the lap-shear test results.

Three lap-shear test averages are displayed in Table 6 following the diffusion bonding. Following the welding, no evidence of plastic deformation was seen in the specimens. In terms of welding temperature and duration, the weakest lap-shear strength was obtained at 850 °C, while the strongest lap-shear strength was acquired at 950 °C, based on lap-shear test values compared to a Ni-Ti6Al4V specimen.

The average of the specimens bonded at 900 °C was used to calculate the lap-shear strength (Table 6). We may conclude that raising the welding temperature will increase the lap-shear strength.

In the lap-shear test of Ti6Al4V/Ni samples, the strongest shear strength was obtained as 244 MPa at a temperature of 950 °C and a time period of 60 min. As shown in Figure 10, the shear strength increased with the increase in temperature. The shear strength of the samples that were bonding at 850 °C were low. The reason for this is the low temperature applied to the samples during assembly. Due to insufficient temperature to cause deformation on the contact surfaces, sufficient contact could not be achieved, and this resulted in the formation of a weak bond in the intermediate region. The shear strength of the samples joined at 900 °C is slightly higher than that of the samples joined at 850 °C. This increase in the shear strength can be explained with the increased temperature that presented high contact surface force and mutual increase in the diffusion amounts of the elements. The reason why higher strength was achieved at 950 °C, compared to other temperatures, can be said to be the increase in temperature, surface deformation, and diffusion. Moreover, the increased incorporation of nickel into silver explains the presence of a silver-rich solid solution for shear deformation and higher nominal nickel concentrations [37].

## 4. Conclusions

We performed the diffusion welding of pure Ni, produced by powder metallurgy technique with Ti6Al4V steel, by using a silver interlayer under an argon-gas-protected atmosphere at 5 MPa constant pressure, at three different (850, 900, 950) °C, with a waiting period of 60 min. The following conclusions can be drawn from the experimental results:

1. Hardness changes in the regions are in parallel with the microstructure. It has been determined that low hardness values are associated with the silver-rich interlayer region.
2. Ag diffusion into the main materials was observed at rates varying between 10 and 40 µm in the samples assembled at 900 and 950 °C. In EDS point analyses, it was generally observed that the diffusion of the Ag interlayer into the main material on the Ti6Al4V side was more intense, and the diffusion of Ni into the Ag interlayer was more intense on the Ni side, and the NiAg phase was also frequent.
3. The highest shear strength value was obtained from the sample joined at the 950 °C test condition. Higher test temperature increased the deformation at contact, and this also increased the amount of diffusion. By increasing the welding temperature, the lap-shear strength can be increased.
4. For all the experiments, the SEM and EDS results show that reliable joints along the interface can be achieved at the process temperature of 950 °C for 60 min under 5 MPa pressure condition. This can be associated with the fact that the phases Ti_92_Ni_33_Al_17_ and Ag_11_Ni_16_ were more common at joints made at 950 °C.
5. As the bonding time increased, due to continuous diffusion and the structural heterogeneity disappeared in the nickel materials obtained by the powder metallurgy method, the pores gradually decreased.

## Figures and Tables

**Table 1:** Chemical contents of Ti6Al4V.

Elements	Ti	Al	V	H (max.%)	Fe (max.)
(at.%)	88.74–91	5.5–6.75	3.5–4.5	0.015	0.25

**Table 2:** Parameters used in the diffusion welding process.

Specimen No	N1	N2	N3
Bonding pressure (MPa)	5	5	5
Bonding temperature (°C)	850	900	950
Holding time (min)	60	60	60

**Table 3:** EDS spot scan results of samples combined at 850 °C and corresponding possible phases.

Test Point	Elemental Ratio (at.%)					Possible Phase
	Al	Ti	V	Ni	Ag	
1	11.08	83.6	4.44	0.61	0.27	Ti_642_Al_151_V_32_Ag_3_
2	7.91	80.44	4.96	0.82	5.87	Ti_156_Al_27_V_9_Ag_5_
3	5.69	72.27	4	1.92	16.11	-
4	0.45	20.31	0.46	13.99	64.8	Ag_58_Ti_41_Ni_23_
5	0.41	1.22	0.53	4.01	93.83	Ag_140_Ni_11_
6	1.9	0.55	0.24	96.91	0.39	Rich in Ni
7	0.6	0.44	0.2	98.38	0.39	Rich in Ni

**Table 4:** EDS spot scan results of samples combined at 900 °C and corresponding possible phases.

Test Point	Elemental Ratio (at.%)					Possible Phase
	Al	Ti	V	Ni	Ag	
8	11.74	84.07	3.27	0.65	0.27	Ti_363_Al_90_V_17_
9	9.56	76.71	5.67	1.37	6.69	-
10	1.49	3.98	0.57	8.69	85.27	Ag_224_Ni_41_Ti_23_
11	0.59	0.8	0.52	8.87	89.22	Ag_11_Ni_2_
12	1.93	2.9	0.52	7.11	87.55	Ag_80_Ni_12_Ti_11_
13	0.22	0.5	0.26	98.56	0.45	Rich in Ni

**Table 5:** EDS spot scan results of samples combined at 950 °C and corresponding possible phases.

Test Point	Elemental Ratio (at.%)					Possible Phase
	Al	Ti	V	Ni	Ag	
14	11.56	81.19	6.04	0.89	0.33	Ti_12_Al_3_V
15	9.95	80.17	6.49	2.94	0.46	Ti_725_Al_147_V_23_
16	8.44	72.19	9.7	5.54	4.13	
17	0.76	1.19	0.56	1.87	95.62	Rich in Ag
18	5.3	64.76	0.9	28.49	0.55	Ti_92_Ni_33_Al_17_
19	3.57	45.45	0.73	40.44	9.81	Ni_16_Ag_11_
20	1.43	2.53	0.43	42.21	53.4	
21	2.11	0.59	0.28	96.51	0.51	Rich in Ni

**Table 6:** Results of the lap-shear test.

Specimens	Temperatures	Durations (min.)	Lap-Shear (MPa)
N1	850 °C	60	193
N2	900 °C	60	238
N3	950 °C	60	244

## Data Availability

The original contributions presented in the study are included in the article, further inquiries can be directed to the corresponding author.
